# A Randomized Study Evaluating the Effect of Ossein–Hydroxyapatite Complex on the Functional Outcomes of Patients After Conservative Treatment of Distal Radius Fracture

**DOI:** 10.3390/ph19060938

**Published:** 2026-06-14

**Authors:** Monika Zaborska, Michał Sobczak, Weronika Kubas, Łukasz Tomczyk, Piotr Morasiewicz

**Affiliations:** 1 Faculty of Medicine, Institute of Medical Sciences, University of Opole, ul. Oleska 48, 45-052 Opole, Poland; 2 Department of Food Safety and Quality Management, Poznan University of Life Sciences, Wojska Polskiego 31, 60-624 Poznań, Poland; 3 Department of Orthopaedic and Trauma Surgery, Institute of Medical Sciences, University of Opole, al. Witosa 26, 45-401 Opole, Poland

**Keywords:** ossein, hydroxyapatite, ossein–hydroxyapatite complex, Osteogenon, OHC, distal radius fractures, muscle strength, wrist mobility, ROM

## Abstract

**Background:** Distal radius fractures (DRFs) are the most common upper limb fractures worldwide. The main goal of DRF treatment is to achieve optimal functional outcomes with the lowest complication rate as rapidly as possible. Achieving full limb function may be delayed by emerging complications or, in some cases, may never occur. Preserving muscle strength and as full a range of motion (ROM) in the wrist as possible are key in DRF management since they enable patients to perform the activities of daily living. The purpose of this study was to assess the effect of ossein–hydroxyapatite complex (OHC), used as an adjunct in conservative DRF treatment, on muscle strength and ROM. **Methods:** This was a prospective randomized clinical study. We assessed 31 patients who underwent non-surgical DRF treatment at our center in the years 2024–2025 and were receiving OHC throughout their fracture treatment. K-Grip and K-Push dynamometers were used to measure the maximum and average muscle strength via tests of grip strength, palmar flexion, and dorsal flexion. Wrist ROM was also evaluated. The results were compared with those of the control group (31 patients receiving DRF treatment without OHC) and with the intact limb. **Results:** The medians of the maximum muscle strength in each test were comparable between the study groups. Both groups showed a higher median average strength in the intact limb than in the treated limb. We observed no intergroup differences in wrist ROM, with ROM parameters lower in the fractured limb than in the intact limb. **Conclusions:** The additional use of OHC was not associated with statistically significant improvements in functional outcomes. The patients from both groups achieved worse muscle strength and ROM outcomes in the fractured than in the intact limb. We recommend a longer and more intense rehabilitation of patients with DRFs. More studies on this topic are needed in order to unequivocally verify the effects of OHC on functional parameters in fracture patients.

## 1. Introduction

Distal radius fractures (DRFs) are the most common upper limb fractures worldwide and are predicted to be even more common in the near future [1,2,3,4]. DRFs are the second most common fracture type in the elderly and constitute approximately 20% of all diagnosed fractures [5]. DRFs are a global problem due to their worldwide prevalence.

A total of 175,040 cases of DRFs were reported the United States in the period 2015–2020 [1]. In the years 2016–2019, DRFs were the cause of 3.6 out of 1000 injury-related visits to the emergency room in the US and constituted 10.8 out of 1000 upper limb injuries overall [2]. In 2022, in Germany, DRF was the third most common fracture necessitating a hospital admission (72,786 cases; prevalence 106/100,000) and the most common fracture treated in an outpatient setting (177,793 cases; prevalence 211/100,000) [3]. Another German study, conducted by Reiland et al., retrospectively assessed a group of 974,332 citizens over 60 years of age who were covered by the mandatory German national health insurance, revealing 17,705 cases of DRFs in that population in the years 2014–2018 [4].

DRFs are common in patients over 50 years of age, with the risk increasing with age. The most common mechanism of fracture in the elderly is low-energy falls from standing height onto an outstretched hand [4,6].

DRFs are associated with pain, limited limb function, and—often—with disability-related absence from work. These fractures may also cause patients stress and financial problems. Due to their high incidence, DRFs pose a burden for healthcare systems [5].

A crucial goal of DRF management is to restore optimal limb function as quickly as possible, with as few complications as possible. Complete restoration of limb function may be delayed due to complications, or—in some cases—may never occur [5,7,8,9].

Improved upper limb function is observed within the first 6 months of fracture [7]. Up to 73% of patients treated for DRF report complications [8]. These are both short-term and long-term complications and functional limitations of the limb. Short-term limitations include limited mobility of the forearm and wrist and a reduced grip strength observed directly after cast removal. Long-term complications, which last even up to 10 years after treatment completion, include persistent pain and wrist ROM deficits, which limit more sophisticated, precise movements [7,8,9]. Moreover, DRFs are associated with the risk of post-traumatic osteoarthritis, complex regional pain syndrome, carpal tunnel syndrome, tendon tears, or bone fragment misalignment, which may considerably limit activities of daily living [7,8,9]. Diminished grip strength and wrist ROM result in difficulties with activities of daily living requiring dexterity and strength, such as getting dressed, showering, cleaning, or cooking. Statistically speaking, approximately 21.2% of patients never regain full limb function [7].

The objectives of fracture treatment are to achieve the best functional outcomes—with the lowest complication rates—over the shortest possible period of time and help the patient regain full limb function. Preserving muscle strength and as good a ROM at the wrist as possible are the key goals of DRF treatment, which help patients in daily functioning and in performing activities of daily living.

The purpose of this study was to assess the effect of ossein–hydroxyapatite complex (OHC), tradename Osteogenon, used as an adjunct during conservative treatment of DRFs on short-term post-treatment limitations: muscle strength and wrist ROM.

Only a limited number of reports have shown beneficial effects of OHC use on clinical and radiological outcomes of fracture treatment, including two retrospective studies conducted by our team [10,11,12]. According to these reports, OHC has a beneficial effect on post-fracture bone remodeling. OHC is the main component of the drug Osteogenon, which has been licensed and marketed for the treatment of osteoporosis and as an adjunct treatment of fractures. Osteogenon contains 830 mg of OHC, including 444 mg of hydroxyapatite, which is equivalent to 178 mg of calcium and 82 mg of phosphorus. The drug provides the structural components essential for bone growth, stimulates osteoblasts, and inhibits osteoclasts. The mechanism of action also includes increased bone metabolism, osteogenesis activation, accelerated callus formation, and an increase in bone mass. The inorganic component of Osteogenon, hydroxyapatite, inhibits bone tissue resorption [10].

Due to the prevalence and incidence of DRFs, the potential improvement and acceleration of treatment through the use of adjunctive pharmacology seems desirable.

As far as we know, there have been no studies assessing the effects of OHC on functional outcomes of fracture treatment. There are no reports on how OHC administration throughout fracture treatment affects muscle strength or wrist ROM. Our study is the first to address this topic in the globally available English-language research literature. The effect of OHC on fracture treatment outcomes has not been thoroughly evaluated or reported.

We conducted this study to test our research hypothesis of potentially beneficial effects of OHC on functional outcomes of DRF treatment, that is, on muscle strength and ROM, thus filling a gap in the global research literature.

## 2. Results

### 2.1. Muscle Strength Measurement Results

The median peak strength was comparable between groups. The median average strength in the intact limb was greater than that in the fractured, treated limb in both groups.

Muscle strength measurement results of the experimental and control group are presented in Table 1. The inter-group differences in these measurements were not statistically significant.

Table 2 shows a comparison between individual muscle strength values measured in the intact and the fractured treated limb in the group of patients receiving OHC—the drug Osteogenon. The inter-limb differences were statistically significant (*p* < 0.05) apart from one parameter—palmar wrist flexion—average strength—for which the *p*-value of 0.0531 showed borderline significance (Table 2).

Table 3 shows a comparison between individual muscle strength values in the intact and fractured limb in the control group. The inter-limb differences in all measurements were statistically significant.

**Figure 1:**
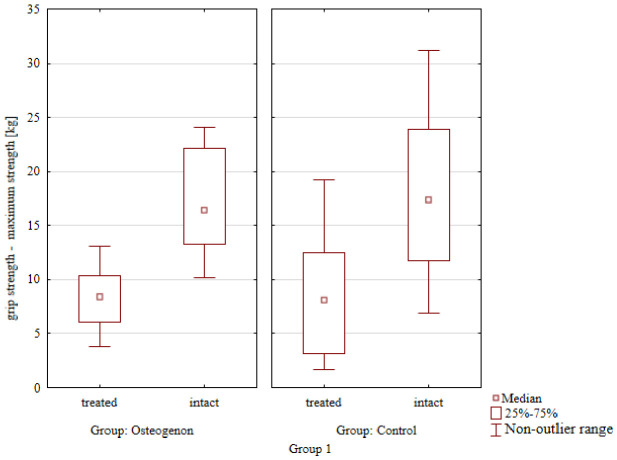
A comparison of the median maximum grip strength between the fractured (treated) and intact (healthy) limb in the experimental group and in the control group.

The median maximum grip strength in patients receiving OHC was 8.40 kg in the fractured limb and 16.45 kg in the intact limb (*p* = 0.0012), whereas in the control group it was 8.15 kg and 17.40 kg, respectively (*p* = 0.0073) (Figure 1).

**Figure 2:**
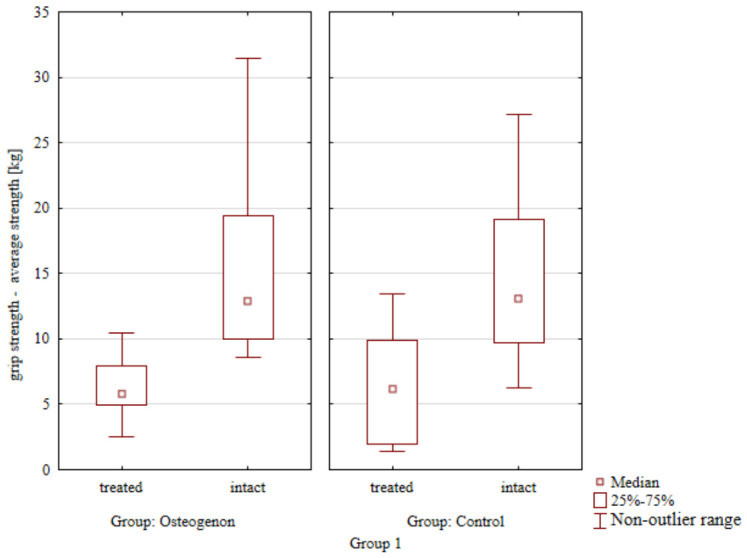
A comparison of the median average grip strength between the fractured (treated) and intact (healthy) limb in the experimental group and in the control group.

The median average grip strength in the OHC group was 5.80 kg in the fractured limb and 12.90 kg in the intact limb (*p* = 0.0014), whereas in the control group it was 6.20 kg and 13.05 kg, respectively (*p* = 0.0029) (Figure 2).

**Figure 3:**
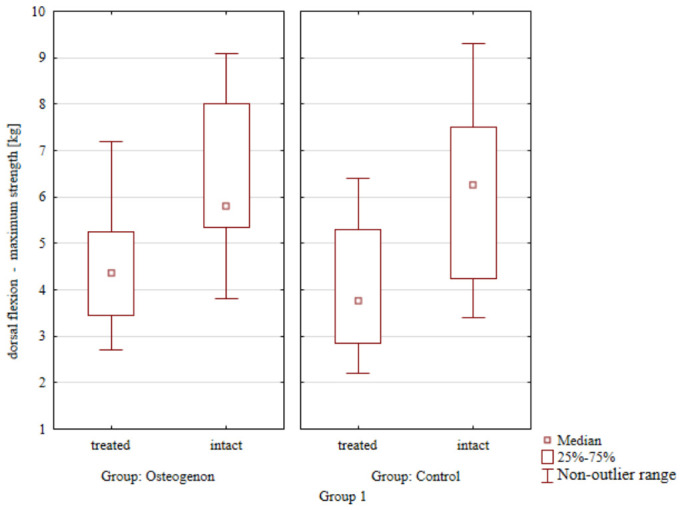
A comparison of the median maximum strength of dorsal wrist flexion between the fractured (treated) and intact (healthy) limb in the experimental group and in the control group.

The median peak strength of dorsal wrist flexion in the patients receiving OHC was 4.35 kg in the fractured limb and 5.80 kg in the intact limb (*p* = 0.012), whereas in the control group it was 3.75 kg and 6.25 kg, respectively (*p* = 0.0194) (Figure 3).

**Figure 4:**
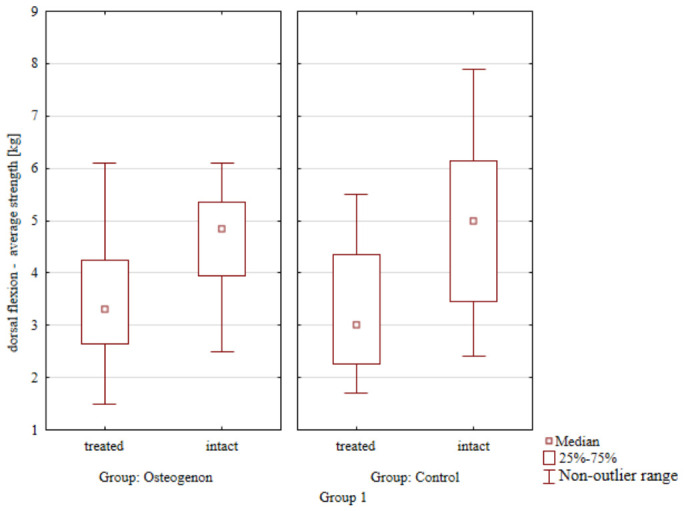
A comparison between the median average strength of dorsal wrist flexion between the fractured (treated) and intact (healthy) limb in the experimental group and in the control group.

The median average strength of dorsal wrist flexion in patients receiving OHC was 3.30 kg in the fractured limb and 4.85 kg in the intact limb (*p* = 0.0194), whereas in the control group it was 3.00 kg and 5.00 kg, respectively (*p* = 0.0226) (Figure 4).

**Figure 5:**
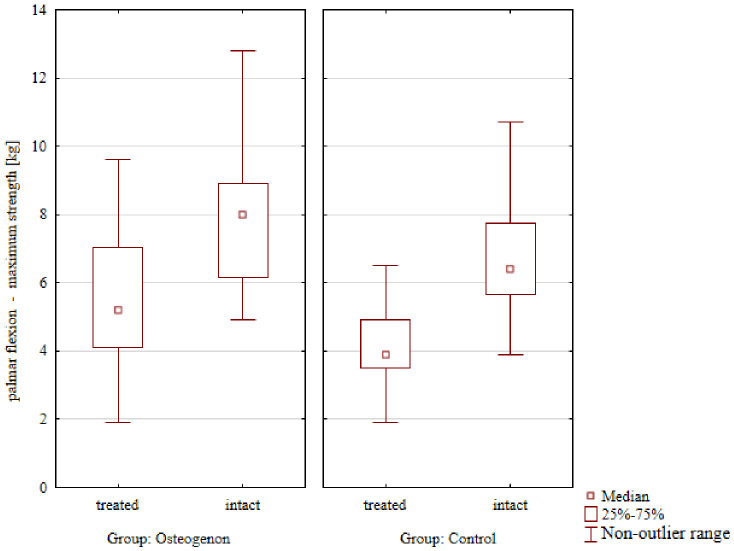
A comparison of the median maximum strength of palmar wrist flexion between the fractured (treated) limb and the intact (healthy) limb in the experimental group and in the control group.

The median maximum strength of palmar wrist flexion in patients receiving OHC was 5.20 kg in the fractured limb and 8.00 kg in the intact limb (*p* = 0.0496), whereas in the control group it was 3.90 kg and 6.40 kg, respectively (*p* = 0.0036) (Figure 5).

The median average strength of palmar wrist flexion in patients receiving OHC was 4.45 kg in the fractured limb and 6.60 kg in the intact limb (*p* = 0.0531), whereas in the control group it was 3.05 kg and 5.45 kg, respectively (*p* = 0.0009).

### 2.2. Range-of-Motion (ROM) Measurement Results

There were no differences between the groups in wrist ROM. In both groups the ROM parameters were lower in the fractured than in the intact limb.

The results of ROM measurements in the experimental and control groups are presented in Table 4. The inter-group differences in these measurements were not statistically significant.

Table 5 presents detailed data on the wrist ROM in the fractured and intact limbs of patients receiving OHC. The inter-limb differences in all measurements were statistically significant.

Table 6 shows detailed data on wrist ROM in the fractured and intact limb in patients from the control group. The inter-limb differences in all measurements were statistically significant.

The median dorsal wrist flexion in the Osteogenon group was 41.5° in the fractured, treated limb and 70.0° in the intact, healthy limb (*p* = 0.0007).

**Figure 6:**
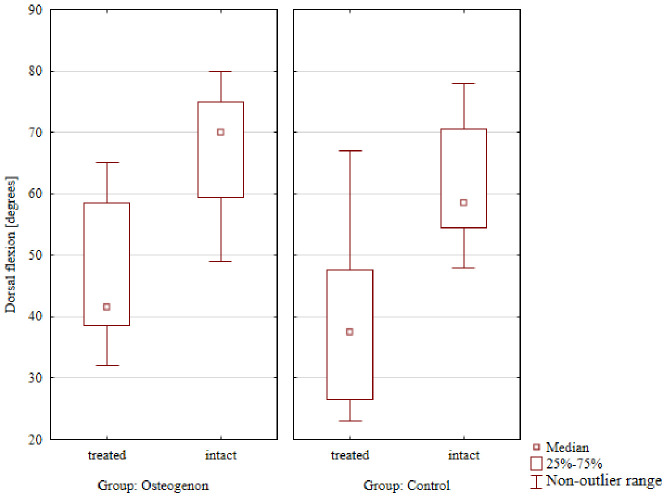
A comparison of the median dorsal wrist flexion between the experimental group and the control group.

The median dorsal wrist flexion in the control group was 37.5° in the treated limb and 58.5° in the intact limb (*p* = 0.0006) (Figure 6).

**Figure 7:**
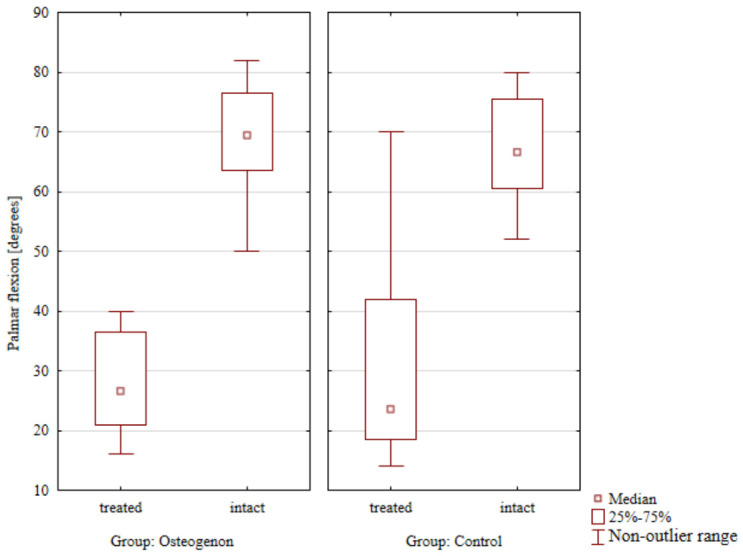
A comparison of the median palmar wrist flexion between the experimental group and the control group.

The median palmar wrist flexion in the Osteogenon group was 26.5° in the treated limb and 69.5° in the intact limb (*p* < 0.0001). The same parameter in the control group was 23.5° in the treated limb and 66.5° in the intact, healthy limb (*p* = 0.0005) (Figure 7).

**Figure 8:**
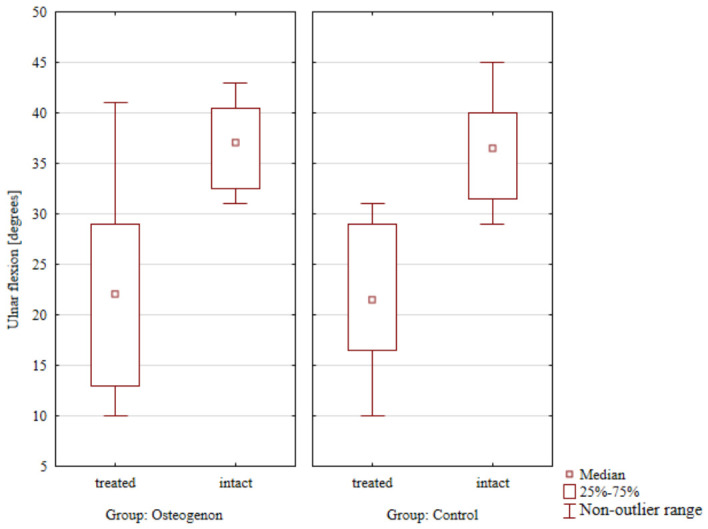
A comparison of the median ulnar wrist flexion between the experimental group and the control group.

The median ulnar wrist flexion in the Osteogenon group was 22.0° in the treated limb and 37.0° in the intact, healthy limb (*p* = 0.0027). The median ulnar wrist flexion in the control group was 21.5° in the treated limb and 36.5° in the intact, healthy limb (*p* = 0.0002) (Figure 8).

**Figure 9:**
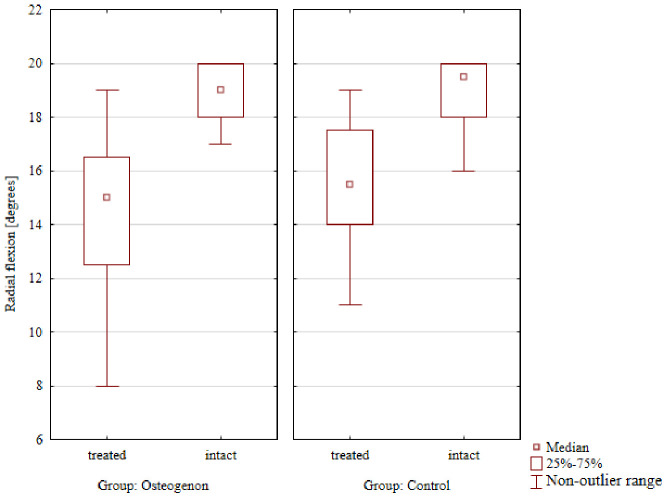
A comparison of the median radial wrist flexion between the experimental group and the control group.

The median radial wrist flexion in the Osteogenon group was 15.0° in the fractured, treated limb and 19.0° in the intact, healthy limb (*p* = 0.0003). The median radial wrist flexion in the control group was 15.5° in the treated limb and 19.5° in the intact, healthy limb (*p* = 0.0012) (Figure 9).

## 3. Discussion

Patients with DRFs need to fully regain their pre-injury muscle strength. Since there was no data on the pre-injury muscle strength in the fractured limb, we used the intact, healthy limb as a point of reference, disregarding the slight differences between the dominant and nondominant limbs [13]. In terms of grip strength and the strength necessary for palmar and dorsal wrist flexion (both maximum and average), our measurements yielded significantly worse results in the fractured limb than in the intact limb, both in the Osteogenon group and in the control group. A comparison between the intact and fractured limb in terms of wrist ROM also showed significantly worse values in the fractured limb in both study groups. In managing limb fractures, it is advisable to help patients quickly achieve limb function comparable to that of the intact limb. Upper limb disability is associated with considerable limitations in patients’ activities of daily living.

The process of regaining pre-DRF function of the injured limb is long and may last up to a decade. Schmidt et al. reported conservatively treated patients with DRF achieving 64%, 89%, and 96% of the grip strength in the intact limb at month 3, year one, and year ten of follow-up [14]. A study by Olech et al. demonstrated that patients with DRFs treated conservatively achieved a mean strength of 76% of that in the intact limb after a mean follow-up of 15 months [15]. Even a slight acceleration of the treatment process would be of considerable benefit for patients, orthopedists, and healthcare systems [5].

The median maximum and average strength achieved in all three tests did not differ significantly between the experimental and control group. The patients receiving OHC failed to achieve significantly better outcomes in terms of muscle strength, which refutes our initial hypothesis of OHC benefits.

A study conducted with 750 participants showed that patients aged 30–49 years have the highest grip strength, which diminishes later with age [13]. The study showed that grip strength outcomes in men were 40% better than those in women [13]. Bobos et al., who evaluated patients with DRFs, reported a mean grip strength of 21.1 kg in men and 8.8 kg in women at month 6 of follow-up [16]. In our study the mean patient age was 68 years, and the vast majority of study participants were women. The mean grip strength in the fractured limb after 6 months of follow-up was 8.4 kg in the OHC group and 8.15 kg in the control group. Klum et al. reported a median grip strength of 20.7 kg in the right hand and 21.0 kg in the left hand in a group of healthy female manual workers aged 50–65 years [13]. In our study, patients achieved a grip strength in the healthy, intact limb of 16.45 kg in the experimental group and 17.4 kg in the control group. Due to the fact that our study population comprised mostly women (whose muscle strength has been shown to be weaker) and elderly patients, our results seem to be consistent with those reported by other authors [13,16,17]. This suggests comparability and reproducibility of our study results. There are no studies in the relevant literature that assess the muscle strength needed for palmar and dorsal flexion of the wrist in patients with a history of DRF. We observed a significant difference between the results achieved in the fractured (treated) and intact (healthy) limb. Patients with DRFs failed to regain full muscle strength by month 6 of follow-up, achieving worse results than in the intact limb. We believe that patients with DRFs require longer and more intensive rehabilitation.

Another objective of DRF management is to achieve the best possible ROM—comparable with population norms for age and sex and with the ROM in the intact limb—within the shortest possible time. In a study conducted in 750 subjects, women achieved better ROM parameters than men [13]. Irrespective of the sex, ROM diminishes with age, with individuals 19–29 years old having the greatest ROM [13].

Olech et al. evaluated ROM in 50 patients with a healed DRF. The mean palmar flexion in the fractured wrist was 67.9° [15]. In two other studies, patients with conservatively treated DRFs achieved 64.8° and 63.0° of palmar flexion [17,18]. In our present study, patients with DRFs achieved a mean palmar flexion of 41.5° in the group receiving OHC and 37.5° in the control group. The mean palmar flexion reported by Kim et al. in a population of 52 healthy isndividuals was 74.2° and that reported by Olech et al. was 84.4° [15]. In our study, patients achieved in the intact limb a mean palmar flexion of 70.0° in the OHC group and 58.5° in the control group. Our patients achieved worse results in terms of dorsal wrist flexion than those reported by other authors. The achieved values are unsatisfactory, with the observed ROM limitations likely associated with limitations in daily living.

Patients treated for DRFs who were evaluated in other studies achieved a mean dorsal wrist flexion of 61°, 61.5°, or 66.5° [15,17,18]. The patients with DRF assessed in our study achieved a mean dorsal flexion of 26.5° in the OHC group and 23.5° in the control group. The mean dorsal wrist flexion in the healthy population has been reported to be 71.1° or 84.5°, depending on the study [15,19]. In our study, mean dorsal wrist flexion in the intact limb was 69.6° in the experimental group and 66.5° in the control group. We observed dorsal flexion limitations in patients after DRF in comparison with the data reported by other authors.

The mean post-DRF radial wrist flexion was 10.0° as reported by Testa et al., 16.0° by Venkatesh et al., and 18.6° by Olech et al. [18,20,21]. In our study, the mean radial flexion in patients after DRF was 15.0° in the experimental group and 15.5° in the control group. The mean radial flexion in healthy limbs was 19.7° as reported by Kim et al. and 27.8° as reported by Olech et al. [15,19], whereas this parameter in our study was 19.0° in the experimental group and 19.5° in the control group. Therefore, the radial flexion results obtained in our study show no significant differences from the data reported in the literature.

The mean ulnar flexion in patients with a healed DRF was 28.2° as reported by Testa et al., 22.0° as reported by Venkatesh et al., and 33.3° as reported by Olech et al. [18,20,21]. The patients in our study achieved a mean ulnar flexion of 22.0° in the Osteogenon group and 21.5° in the control group. The mean values of ulnar wrist flexion in intact limbs reported in the literature were 34.0° and 45.4° [15,19]. The patients evaluated in our study achieved a mean ulnar wrist flexion of 37.0° in the Osteogenon group and 36.5° in the control group. The results of patients in our study are comparable with those reported by other authors.

Out of the two types of functional parameters assessed in the injured limb of patients with DRF—ROM and muscle strength—ROM reached values comparable with those in the intact limb faster. In a study by Schmidt et al., conservatively treated patients with DRFs achieved 86% of the ROM of the intact limb at month three, 94% at year one, and 96% at year ten after treatment [14]. The radial and ulnar wrist flexion values achieved in our study, both in the Osteogenon and the control group, are comparable with those reported in the global scientific literature. In our study, both palmar and dorsal wrist flexion in the treated limb were worse than in the intact limb. In order to achieve ROM norms for age and sex, and to regain full limb function, patients with DRFs require further rehabilitation.

DRF constitutes an estimated 18% of all fractures in patients older than 65 years, who are referred to as geriatric patients in the American Academy of Orthopedic Surgeons Appropriate Use Criteria for the treatment of DRFs. At that age, these fractures are much more common in women, whose estimated lifetime risk of DRFs is 15%, with the incidence of DRFs in women between 60 and 99 years of age nearly threefold higher. The lifetime risk of DRF in men is 2%. Falls and balance problems, collectively referred to as “instability,” belong to the so-called geriatric giants. Statistically, one in three individuals over the age of 65 years experiences falls, which is the sixth most common cause of death in people over 65 years old. Falls in women are 30% more common than in men [20,21]. The vast majority of patients in our study were women, and the mean age was 68 years, which is consistent with data from the literature.

The use of adjunctive devices or medications plays an important role in orthopedics in terms of clinical improvement [22]. In the case of DRFs, there are studies regarding the use of pharmacotherapy as an adjunct to non-surgical treatment with vitamin D3 and teriparatide.

A total of 32 postmenopausal women with a conservatively treated distal radius fracture participated in a study by Heyer et al. examining the effects of vitamin D^3^ on the treatment of DRF [23]. Ultimately, 10 women were randomized to supplement high-dose vitamin D^3^ (1800 IU per day), 10 women were randomized to supplement low-dose vitamin D^3^ (700 IU per day), and 10 women were included into the control group (no supplementation). This study did not demonstrate a positive effect of vitamin D^3^ supplementation on the outcome of DRF treatment. No statistical differences were observed between the low-dose vitamin D^3^ supplementation group and the control group, while the high-dose supplementation group demonstrated a reduced trabecular number and lower compressive stiffness [23]. The authors assessed the effect of vitamin D^3^ on the functional outcomes of DRF treatment using a standardized Patient-Rated Wrist Evaluation (PRWE) questionnaire. No differences were observed between the vitamin D^3^ supplementation group and the control group [23].

In a study by Aspenberg et al., 102 postmenopausal women were randomly assigned to three groups, each containing 34 patients: a group receiving teriparatide 40 µg, a group receiving teriparatide 20 µg, and a placebo group [24]. In this study, no statistically significant difference was found between the 40 µg teriparatide group and the placebo group, or between the 40 µg teriparatide group and the 20 µg teriparatide group. A shorter healing time was observed in the group receiving teriparatide at a dose of 20 µg compared to the group without intervention [24]. The researchers also examined how additional teriparatide use would affect grip strength. No statistically significant differences in limb function were observed between the study and control groups [24].

The use of adjunct medical treatment in the form of OHC throughout the fracture treatment period in our study did not affect patients’ muscle strength or ROM outcomes. The goal of DRF management is to achieve parameters comparable to those in the intact limb during the possibly shortest convalescence period, which enables the patient to resume their daily activities. In a younger age group, working patients would be able to reduce their absences and quickly resume work. In the elderly, resuming everyday activities is also very important, since regular physical activity 2–3 times a week reduces the risk of falls [20]. Crandall et al. showed that women—particularly those aged ≥ 80 years—were much more likely to experience deterioration in their fitness levels within 5 years following a wrist or forearm fracture in comparison with women with no fracture history [25]. As a result, it is crucial for injured patients to resume their daily activities as soon as possible, since this reduces their risk of another fall.

Diminished physical activity as a result of a fracture or treatment complications not only considerably lowers the patient’s quality of life, but can also increase the risk of sarcopenia in geriatric patients. Concomitant sarcopenia and osteoporosis—referred to as osteosarcopenia—pose a considerable burden for the musculoskeletal system, negatively affect the patient’s wellbeing, mobility, and general quality of life, and increase the risk of falls and fractures (particularly fractures of the hip, vertebrae, and wrist). A systematic review by Veronese et al. demonstrated that osteosarcopenia increases the risk of death by approximately 53% [26]. Regular physical activity at an older age has a beneficial effect on cognitive function, by preventing cognitive decline and slowing down the development of such conditions as Alzheimer disease [27].

There are scientific reports suggesting that earlier initiation of rehabilitation may have a positive effect on the functional outcomes of treatment. Nguyen et al. conducted a randomized controlled trial to examine how a proprietary exercise program focused on hand strength affects grip strength in patients over 60 years of age with non-surgically treated DRFs. Patients in the study group performed exercises affecting the range of motion of the fingers using a rubber stress ball for 2 to 6 weeks after the injury, while immobilized in a full short cast. After the cast was removed, patients in both groups received the same standard rehabilitation recommendations. After 6 and 12 weeks, grip strength was assessed using a dynamometer. Patients in the study group who performed the exercises showed statistically significantly better results than the control group (81% vs. 51% after 12 weeks) [28].

In order to ensure optimal treatment outcomes for the patient’s benefit, it is necessary to optimize the treatment protocol. Thus, in the case of patients with a recent history of DRF—due to the long period of convalescence—it is advisable to prolong and intensify the rehabilitation regime until satisfactory treatment outcomes (comparable with those in the intact limb) have been achieved.

## 4. Materials and Methods

This was a prospective randomized study (retrospectively registered on ClinicalTrials.gov; NCT07210281) conducted to assess functional outcomes of DRF treatment—muscle strength and wrist ROM—in patients treated at the University Teaching Hospital in Opole in Poland during the period from 2024 to 2025 and receiving OHC (Osteogenon) throughout treatment duration. The study was designed as a randomized clinical trial with parallel groups and was exploratory in nature.

Participants of this study had to have met the following inclusion criteria:

- A conservatively treated fracture of the distal radius;
- Age between 18 and 85 years;
- A written informed consent;
- No contraindications to taking Osteogenon (such as hypersensitivity to the active ingredient or to any of the excipients, severe renal failure and dialysis therapy, hypercalcemia, hypercalciuria, calcium-based kidney stones, or tissue calcifications);
- Not taking any other medications that could affect bone tissue remodeling (such as those used in osteoporosis therapy);
- No other limb pathologies (inflammatory or neurogenic), and no comorbidities that could affect bone union;
- Not taking other medications that interact with Osteogenon (e.g., thiazide diuretics).

The same inclusion criteria were applied to patients in the experimental group enrolled at a later stage and also to those randomized into the control group. The participants in both groups were recruited (from 7 October 2024 to 31 January 2025) from among the patients who presented at the emergency room of our center with a DRF diagnosed based on X-rays or computed tomography.

In the study, randomization was stratified by age (≤60/>60 years) and gender (women/men), with blocks of varying sizes (2, 4, 8 people) carefully generated manually. A separate allocation sequence was prepared for each stratum, and Sequentially Numbered, Opaque, Sealed Envelopes (SNOSE) were used to assign participants to individual groups.

While the patients were not blinded to group allocation, members of the research team evaluating the results were. The data used for the statistical analysis of the results were fully anonymized, with no data allowing the identification of group allocation.

Over the study accrual period, our center received 163 patients with DRFs, including 126 adults, 84 of whom met the study inclusion criteria. These eligible patients were divided into experimental and control groups by stratified block randomization (blocks of 2, 4, and 8 individuals). Both study groups comprised 42 participants.

Fractures in patients from both groups were managed following the same treatment protocol, which involved fracture reduction and limb immobilization with a forearm cast. The patients received the same rehabilitation recommendations and underwent the same clinical and radiological follow-up protocol.

Immediately after closed DRF reduction and cast immobilization, a follow-up X-ray was obtained in the emergency department. If the bone fragments were properly aligned, the patient was discharged from the hospital and scheduled for an outpatient follow-up visit (physical examination and radiography) within 5–7 days of injury. During the initial follow-up visit in the orthopedic clinic, patients were prescribed hand and elbow exercises (following the same protocol) and instructed in their performance. Subsequent follow-up visits took place 7 days, 14 days, and 4–5 weeks after the injury. After cast removal, patients were once again prescribed hand, wrist, and elbow exercises (following the same protocol). Subsequent follow-up visits (physical examination and radiography) took place 7 days, 3 weeks, and 6 weeks after cast removal. Any subsequent follow-up visits, if necessary, were held at three-month intervals. Patients were encouraged to perform home exercises daily. During scheduled follow-up visits, patients were asked about their regular adherence to the exercise program and this was recorded in their medical records. Patients were instructed to perform the exercises until a follow-up visit scheduled 6 weeks after the cast was removed.

OHC-group patients received two tablets of Osteogenon per day (one tablet every 12 h) throughout the treatment period. One tablet of the drug contains 830 mg of OHC, which includes 444 mg of hydroxyapatite—equivalent to 178 mg of calcium and 82 mg of phosphorus. Each follow-up visit involved questions about regular use of the study drug, with additional packs prescribed as needed. Patients from the control group received neither Osteogenon nor placebo. We had complete medical records of all the patients at our disposal.

**Figure 10:**
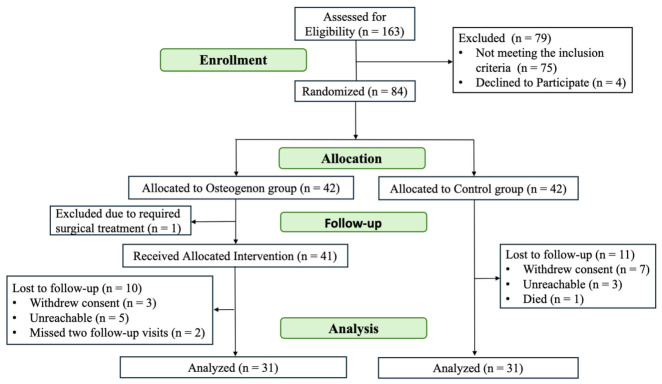
CONSORT flow diagram. Diagram showing eligible, recruited, observed, and analyzed patients.

Study participation was voluntary, with the option of withdrawing from the study at any time. Out of the 84 patients initially deemed eligible for the study, 1 female was excluded due to the need for surgical treatment. Once bone union was achieved, ten patients withdrew their consent for further participation, eight patients proved unreachable, two patients missed two scheduled follow-up visits despite previous phone confirmation, and one patient died (Figure 10). Ultimately, data from 62 cases were included in our analysis (Figure 10). The experimental group ultimately comprised 31 patients at a mean age of 68 years (41–82 years), and the control group comprised 31 patients at a mean age of 67 years (47–87).

The Osteogenon and control groups showed no significant differences in terms of mean body mass index (BMI), distribution of fracture types (AO/OTA fracture classification), age, sex, or the proportion of smokers (Table 7).

Muscle strength and ROM were assessed at least six months after fracture. The median follow-up period in the Osteogenon group was 137 days (91–194 days). The two groups did not differ in terms of follow-up duration (*p* = 0.949) (Table 7).

**Figure 11:**
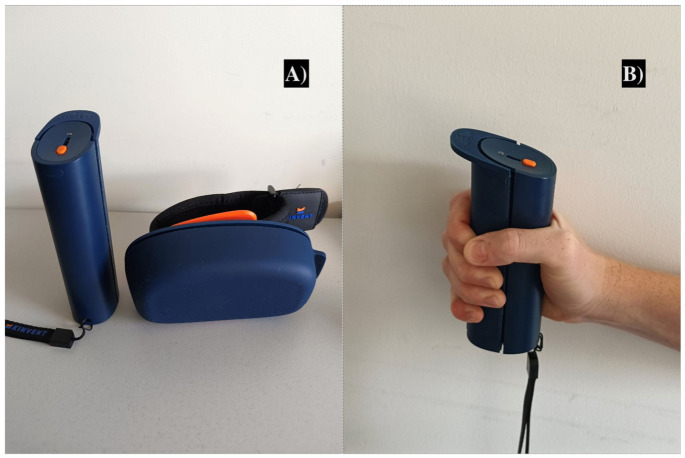
(**A**) K-push and K-grip dynamometers from Kinvent; (**B**) grip strength assessment with a K-grip device.

Muscle strength was measured with K-Grip (KFORCEGrip) and K-Push (KFORCEMuscle) devices (Kinvent). These are portable, wireless dynamometers, which connect via Bluetooth with a laptop, cell phone, or tablet, with an installed Kinvent application (Figure 11).

Muscle strength at the wrist was assessed by measuring grip strength and the strength of dorsal and palmar wrist flexion. The following parameters were measured in the fractured and intact limb: maximum strength [expressed in kg], average strength [expressed in kg], inter-limb maximum strength asymmetry [expressed in %], and inter-limb average strength asymmetry [expressed in %]. Each patient underwent these measurements three times, with the mean values used in further analysis. Maximum strength represented the maximum value in a given limb achieved by the patient in all attempts. Average strength was the average value measured during the attempt that produced the maximum strength. Maximum-strength and average-strength asymmetry between the limbs was the measure of discrepancy between the measured values obtained in the treated limb and in the intact limb.

**Figure 12:**
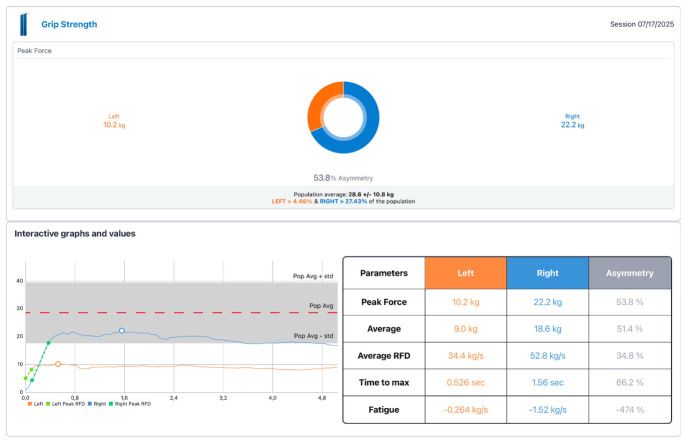
A sample result of grip strength measurement generated by the system; RFD—rate of force development.

A sample report generated by the system is presented below (Figure 12).

Wrist ROM was measured in four directions: radial flexion, ulnar flexion, dorsal flexion, and palmar flexion. Radial wrist flexion is a frontal movement that involves bending the hand toward the radius. Ulnar wrist flexion is the opposite movement, bending the hand toward the ulna. Palmar flexion of the wrist is a movement that occurs in the sagittal plane, involving the palmar surface of the hand approaching the palmar surface of the forearm. Dorsal flexion of the wrist is the opposite movement, involving the dorsum of the hand approaching the dorsal surface of the forearm. ROM was measured with a flexible, full-circle goniometer with two 15 cm arms. This tool was positioned dorsal to the third metacarpal bone, and the angle values were read directly on the goniometer [13]. In order to make them more objective, all measurements were performed by two experienced investigators, with the presented results being the mean values.

We set grip strength in terms of maximum and average strength as the primary endpoint of the study. Range of motion (ROM) measured in various ranges of motion along with dorsal and palmar flexion strength were defined as secondary outcomes.

### 4.1. Statistical Analysis

Statistical analyses were performed using Statistica version 14.1 (TIBCO Software Inc., San Ramon, CA, USA). Data distribution was assessed for normality using the Shapiro–Wilk test. Because most continuous variables did not follow a normal distribution, non-parametric methods were primarily applied. Continuous variables are presented as medians with first (Q1) and third (Q3) quartiles. Between-group comparisons were performed using the Mann–Whitney U test.

Categorical variables are reported as frequencies and percentages and were compared between groups using the chi-square (χ^2^). In addition to *p*-values, effect sizes were calculated for non-parametric comparisons using effect size r (r = |Z|/√N), where Z is the standardized test statistic and N is the total number of observations. To facilitate clinical interpretation, an approximate conversion to Cohen’s d was also provided (d = 2r/√(1 − r^2^)), with the understanding that this approximation should be interpreted with caution in non-parametric analyses.

All statistical tests were two-tailed, and the level of statistical significance was set at *p* < 0.05.

Potential confounding factors, such as age, sex, BMI, and smoking status, were controlled for through stratified block randomization at the stage of participant allocation, to ensure comparable baseline characteristics between the Osteogenon and control groups. Baseline comparability was verified statistically, with no significant intergroup differences observed.

### 4.2. Ethics

The drug Osteogenon used in our study had been approved for fracture treatment. The study had been approved by the local ethics committee at the Lower Silesian Medical Chamber in Wrocław (protocol code 2/PNDR/2020, date of approval 10 June 2020). The study was conducted in accordance with the Declaration of Helsinki and all applicable laws and regulations. All patients provided their written informed consent and were informed of the voluntary nature of their participation in this study. Study participants had the option of withdrawing their consent at any time. This is an investigator-initiated research study with no commercial sponsorship. The study was retrospectively registered on ClinicalTrials.gov (ID: NCT07210281). Registration was terminated after study initiation due to an administrative oversight. The study protocol, eligibility criteria, interventions, and predefined primary and secondary endpoints were established before recruitment began, and no significant modifications were made to the protocol after study initiation. Consolidated Standards of Reporting Trials (CONSORT) guidelines were followed for the preparation of this manuscript [Table S1].

## 5. Limitations

Our study has certain limitations, one of which is the relatively small study population. This resulted from our restrictive study inclusion criteria, which had to be met by patients from both the experimental and control group in order to ensure study result reliability. Another limitation was the list of contraindications for the use of Osteogenon provided by the manufacturer. Additionally, several patients withdrew their consent, were lost to follow-up, proved unreachable, or died. However, other studies on the effects of OHC on fracture treatment were conducted in comparable, if not smaller, populations [10,11,12,13]. Another limitation is the fact that pre-injury wrist muscle strength and ROM were unknown. Therefore, the evaluated parameters of the treated wrist were compared with those of the intact wrist. The proportion of men in our study population was relatively low, whereas the mean age was relatively high. Nonetheless, this pattern is consistent with the epidemiologic data reported in the literature on DRF incidence in adults [4,6,20,21]. Another limitation is the fact that we did not perform an a priori sample size calculation because this study was designed as an exploratory randomized clinical trial. Recruitment was limited by strict eligibility criteria and the practical challenges associated with conducting a prospective clinical study with follow-up assessments in fracture patients. Therefore, we cannot claim that the study was adequately powered to detect small or moderate clinically meaningful differences in grip strength or wrist ROM. To facilitate interpretation of the findings, we reported effect size measures (r and the corresponding approximate Cohen’s d values) for all comparisons. These estimates may serve as a basis for sample size calculations in future adequately powered confirmatory trials. Another limitation is that this was not a placebo-controlled study. Although the study was not placebo-controlled, the assessment of the primary outcome was based on objective instrumental measurements rather than on subjective patient-reported outcomes. Furthermore, the independent statistician analyzing the data was unaware of the group allocation, and patients were treated according to the same protocol, receiving the same rehabilitation recommendations, which may have reduced the risk of expectation and performance bias. The chief advantage of our study is the fact that this topic has not been thoroughly discussed in the literature. This study expands on the adopted research hypothesis of beneficial effects of OHC on fracture healing. As far as we know, none of the available studies evaluated the effects of OHC on the functional outcomes of DRF treatment. Further advantages of our study were its prospective nature, randomization, the use of a consistent treatment and rehabilitation protocol, involvement of experienced orthopedic surgeons, regular follow-up visits, and the use of specialist dynamometers to objectify measurements.

There is a need for further, optimally multicenter, studies in a larger patient population and a longer follow-up, to unequivocally verify the research hypothesis. We are thus planning to conduct a study with a longer follow-up, higher number of patients, and more assessed parameters.

## 6. Conclusions

The endpoints specified in the study did not reach statistical significance. The additional use of OHC, in conjunction with routine, to common DRF treatment protocols was not associated with statistically significant improvements in functional outcomes, including muscle strength and ROM, in this exploratory study.

Patients from both study groups achieved worse muscle strength and ROM outcomes in the fractured limb than in the intact limb.

Patients with DRF require longer and more intensive rehabilitation than that used in current practice.

More studies on this topic are needed in order to unequivocally verify the effects of OHC on functional parameters in fracture patients.

## Figures and Tables

**Table 1:** A detailed muscle strength assessment in patients from the Osteogenon group and the control group.

Analyzed Variable	Group	Q1	Median	Q3	Z	*p*-Value	Effect Size (r)
Maximum grip strength, treated limb [kg]	Osteogenon	6.10	8.40	10.40	0.462	0.644	0.094
	Control	3.20	8.15	12.50			
Average grip strength, treated limb [kg]	Osteogenon	4.95	5.80	7.95	0.404	0.686	0.083
	Control	2.00	6.20	9.85			
Maximum grip strength, intact limb [kg]	Osteogenon	13.25	16.45	22.15	0.000	1.000	0.000
	Control	11.75	17.40	23.85			
Average grip strength, intact limb [kg]	Osteogenon	9.95	12.90	19.45	0.231	0.817	0.047
	Control	9.70	13.05	19.10			
Grip strength asymmetry—maximum strength [%]	Osteogenon	30.71	43.85	67.04	−0.751	0.453	0.153
	Control	31.37	55.89	79.07			
Grip strength asymmetry—average strength [%]	Osteogenon	32.75	49.54	70.81	−0.808	0.419	0.165
	Control	33.53	56.33	81.23			
Dorsal flexion, treated limb—maximum strength [kg]	Osteogenon	3.45	4.35	5.25	0.808	0.419	0.165
	Control	2.85	3.75	5.30			
Dorsal flexion, treated limb—average strength [kg]	Osteogenon	2.65	3.30	4.25	0.404	0.686	0.083
	Control	2.25	3.00	4.35			
Dorsal flexion, intact limb—maximum strength [kg]	Osteogenon	5.35	5.80	8.00	0.202	0.840	0.041
	Control	4.25	6.25	7.50			
Dorsal flexion, intact limb—average strength [kg]	Osteogenon	3.95	4.85	5.35	−0.289	0.773	0.059
	Control	3.45	5.00	6.15			
Dorsal flexion asymmetry—maximum strength [%]	Osteogenon	18.28	26.58	43.25	−0.491	0.624	0.100
	Control	18.60	30.39	41.93			
Dorsal flexion asymmetry—average strength [%]	Osteogenon	10.05	26.78	44.10	−0.462	0.644	0.094
	Control	11.76	29.96	48.64			
Palmar flexion, treated limb—maximum strength [kg]	Osteogenon	4.10	5.20	7.05	1.501	0.133	0.306
	Control	3.50	3.90	4.90			
Palmar flexion, treated limb—average strength [kg]	Osteogenon	3.40	4.45	6.00	1.674	0.094	0.342
	Control	2.70	3.05	4.20			
Palmar flexion, intact limb—maximum strength [kg]	Osteogenon	6.15	8.00	8.90	0.866	0.386	0.177
	Control	5.65	6.40	7.75			
Palmar flexion, intact limb—average strength [kg]	Osteogenon	5.3	6.60	7.45	0.866	0.386	0.177
	Control	4.65	5.45	7.10			
Palmar flexion asymmetry—maximum strength [%]	Osteogenon	7.78	22.71	40.17	−1.530	0.126	0.312
	Control	23.18	38.10	49.25			
Palmar flexion asymmetry—average strength [%]	Osteogenon	4.41	16.56	46.06	−1.761	0.078	0.359
	Control	25.10	44.02	51.97			

Z—Standardized value of the Mann–Whitney U test (with continuity correction); *p*—*p*-value; Q1, Q3—1st and 3rd quartile; r—effect size for the Mann–Whitney test (r = |Z|/√N), N = 24.

**Table 2:** A detailed muscle strength assessment in patients from the Osteogenon group.

Analyzed Variable	Limb	Q1	Median	Q3	Z	*p*-Value	Effect Size (r)
Maximum grip strength [kg]	Treated	6.10	8.40	10.40	−3.233	0.001	0.660
	Intact	13.25	16.45	22.15			
Average grip strength [kg]	Treated	4.95	5.80	7.95	−3.204	0.001	0.654
	Intact	9.95	12.90	19.45			
Dorsal flexion—maximum strength [kg]	Treated	3.45	4.35	5.25	−2.511	0.012	0.513
	Intact	5.35	5.80	8.00			
Dorsal flexion—average strength [kg]	Treated	2.65	3.30	4.25	−2.338	0.019	0.477
	Intact	3.95	4.85	5.35			
Palmar flexion—maximum strength [kg]	Treated	4.10	5.20	7.05	−1.963	0.050	0.401
	Intact	6.15	8.00	8.90			
Palmar flexion—average strength [kg]	Treated	3.40	4.45	6.00	−1.934	0.053	0.395
	Intact	5.30	6.60	7.45			

Z—Standardized value of the Mann–Whitney U test (with continuity correction); *p*—*p*-value; Q1, Q3—1st and 3rd quartile; r—effect size for the Mann–Whitney test (r = |Z|/√N), N = 24.

**Table 3:** A detailed muscle strength assessment in patients from the control group.

Analyzed Variable	Limb	Q1	Median	Q3	Z	*p*-Value	Effect Size (r)
Maximum grip strength [kg]	Treated	3.20	8.15	12.50	−2.685	0.007	0.548
	Intact	11.75	17.40	23.85			
Average grip strength [kg]	Treated	2.00	6.20	9.85	−2.973	0.003	0.607
	Intact	9.70	13.05	19.10			
Dorsal flexion—maximum strength [kg]	Treated	2.85	3.75	5.30	−2.338	0.019	0.477
	Intact	4.25	6.25	7.50			
Dorsal flexion—average strength [kg]	Treated	2.25	3.00	4.35	−2.338	0.023	0.466
	Intact	3.45	5.00	6.15			
Palmar flexion—maximum strength [kg]	Treated	3.50	3.90	4.90	−2.916	0.004	0.595
	Intact	5.65	6.40	7.75			

Z—Standardized value of the Mann–Whitney U test (with continuity correction); *p*—*p*-value; Q1, Q3—1st and 3rd quartile; r—effect size for the Mann–Whitney test (r = |Z|/√N), N = 24.

**Table 4:** A detailed assessment of the range of motion in patients from the Osteogenon group and the control group.

Analyzed Variable	Group	Q1	Median	Q3	Z	*p*-Value	Effect Size (r)
Dorsal flexion, treated limb [degrees]	Osteogenon	38.50	41.50	58.50	−1.415	0.157	0.289
	Control	26.50	37.50	47.50			
Dorsal flexion, intact limb [degrees]	Osteogenon	59.50	70.00	75.00	−1.270	0.204	0.259
	Control	54.50	58.50	70.50			
Palmar flexion, treated limb [degrees]	Osteogenon	21.00	26.50	36.50	−0.520	0.603	0.106
	Control	18.50	23.50	42.00			
Palmar flexion, intact limb [degrees]	Osteogenon	63.50	69.50	76.50	−0.548	0.583	0.112
	Control	60.50	66.50	75.50			
Ulnar flexion, treated limb [degrees]	Osteogenon	13.00	22.00	29.00	−0.029	0.977	0.006
	Control	16.50	21.50	29.00			
Ulnar flexion, intact limb [degrees]	Osteogenon	32.50	37.00	40.50	−0.231	0.817	0.047
	Control	31.50	36.50	40.00			
Radial flexion, treated limb [degrees]	Osteogenon	12.50	15.00	16.50	0837	0.403	0.171
	Control	14.00	15.50	17.50			
Radial flexion, intact limb [degrees]	Osteogenon	18.00	19.00	20.00	0.289	0.773	0.059
	Control	18.00	19.50	20.00			

Z—Standardized value of the Mann–Whitney U test (with continuity correction); *p*—*p*-value; Q1, Q3—1st and 3rd quartile; r—effect size for the Mann–Whitney test (r = |Z|/√N), N = 24.

**Table 5:** A detailed assessment of the range of motion in patients from the Osteogenon group.

Analyzed Variable	Limb	Q1	Median	Q3	Z	*p*-Value	Effect Size (r)
Dorsal flexion [degrees]	Treated	38.50	41.50	58.50	−3.406	<0.001	0.695
	Intact	59.50	70.00	75.00			
Palmar flexion [degrees]	Treated	21.00	26.50	36.50	−4.070	<0.001	0.831
	Intact	63.50	69.50	76.50			
Ulnar flexion [degrees]	Treated	13.00	22.00	29.00	−3.002	0.003	0.613
	Intact	32.50	37.00	40.50			
Radial flexion [degrees]	Treated	12.50	15.00	16.50	−3.608	<0.001	0.737
	Intact	18.00	19.00	20.00			

Z—Standardized value of the Mann–Whitney U test (with continuity correction); *p*—*p*-value; Q1, Q3—1st and 3rd quartile; r—effect size for the Mann–Whitney test (r = |Z|/√N), N = 24.

**Table 6:** A detailed assessment of the range of motion in patients from the control group.

Analyzed Variable	Limb	Q1	Median	Q3	Z	*p*-Value	Effect Size (r)
Dorsal flexion [degrees]	Treated	26.50	37.50	47.50	−3.435	<0.001	0.701
	Intact	54.50	58.50	70.50			
Palmar flexion [degrees]	Treated	18.50	23.50	42.00	−3.493	<0.001	0.713
	Intact	60.50	66.50	75.50			
Ulnar flexion [degrees]	Treated	16.50	21.50	29.00	−3.724	<0.001	0.760
	Intact	31.50	36.50	40.00			
Radial flexion [degrees]	Treated	14.00	15.50	17.50	−3.233	0.001	0.660
	Intact	18.00	19.50	20.00			

Z—Standardized value of the Mann–Whitney U test (with continuity correction); *p*—*p*-value; Q1, Q3—1st and 3rd quartile; r—effect size for the Mann–Whitney test (r = |Z|/√N), N = 24.

**Table 7:** Detailed demographic data of patients from the Osteogenon group and control group.

Analyzed Variable	Group	Q1	Median	Q3	% of Observations	Z/χ^2^	*p*	Effect Size
Age [years]	Osteogenon	62	68	77	-	Z = 0.092	0.927	r = 0.012
	Control	64	69	73	-			
Follow-up [days]	Osteogenon	91	137	194	-	Z = −0.063	0.949	r = 0.008
	Control	97	135	193	-			
BMI [kg/m^2^]	Osteogenon	22.87	26.79	31.17	-	Z = −0.444	0.657	r = 0.056
	Control	22.87	28.19	31.90	-			
Smokers	Osteogenon	-	-	-	9.68	χ^2^ = 0.000	1.000	φ = 0.000
	Control	-	-	-	9.68			
AO/OTA classification—2R3A2.2	Osteogenon	-	-	-	32.26	χ^2^ = 1.915	0.751	V = 0.176
	Control	-	-	-	22.58			
AO/OTA classification—2R3A2.1	Osteogenon	-	-	-	32.26			
	Control	-	-	-	29.03			
AO/OTA classification—2R3A2.3	Osteogenon	-	-	-	16.13			
	Control	-	-	-	16.13			
AO/OTA classification—2R3B3.1	Osteogenon	-	-	-	12.90			
	Control	-	-	-	25.81			
AO/OTA classification—2R3C1	Osteogenon	-	-	-	6.45			
	Control	-	-	-	6.45			

Z—Standardized value of the Mann–Whitney U test; χ^2^—chi-square test statistic; *p*—*p*-value; Q1, Q3—1st and 3rd quartile; r—effect size for the Mann–Whitney test (r = |Z|/√N); φ—effect size for the chi-square test (2 × 2 tables); V—Cramér’s V for contingency tables larger than 2 × 2.

## Data Availability

The raw data supporting the conclusions of this article will be made available by the authors on request.

## Supplementary Materials

The following supporting information can be downloaded at https://www.mdpi.com/article/10.3390/ph19060938/s1, Table S1: CONSORT 2025 checklist of information to include when reporting a randomised trial. Reference [29] is cited in the Supplementary Materials.

## References

[1] Miskiewicz M., Hakimi A., Ling K., Tesoriero J., Komatsu D., Wang E. (2025). Epidemiology, Risk Factor, and Economic Analysis of Peripheral Nerve Injury Following Distal Radius Fractures. Eur. J. Orthop. Surg. Traumatol..

[2] Chinta S.R., Cassidy M.F., Tran D.L., Brydges H.T., Ceradini D.J., Bass J.L., Agrawal N.A. (2024). Epidemiology of Distal Radius Fractures: Elucidating Mechanisms, Comorbidities, and Fracture Classification Using the National Trauma Data Bank. Injury.

[3] Walter N., Szymski D., Bärtl S., Biehl C., Knapp G., Lang S., Alt V., Heiß C., Rupp M. (2025). Prevalence of Fractures in the Adult Population of Germany. Dtsch. Arztebl. Int..

[4] Reiland K., Haastert B., Arend W., Klüppelholz B., Windolf J., Icks A., Thelen S., Andrich S. (2024). Epidemiology of Distal Radius Fractures in Germany—Incidence Rates and Trends Based on Inpatient and Outpatient Data. Osteoporos. Int..

[5] American Academy of Orthopaedic Surgeons (2020). Management of Distal Radius Fractures Evidence-Based Clinical Practice Guideline.

[6] Thorninger R., Romme K.L., Wæver D., Henriksen M.B., Tjørnild M., Lind M., Rölfing J.D. (2023). Posttraumatic Arthritis and Functional Outcomes of Nonoperatively Treated Distal Radius Fractures after 3 Years. Sci. Rep..

[7] Van Son M.A.C., De Vries J., Roukema J.A., Den Oudsten B.L. (2013). Health Status and (Health-Related) Quality of Life during the Recovery of Distal Radius Fractures: A Systematic Review. Qual. Life Res..

[8] Marchewka J., Marchewka W., Golec E. (2021). Quality of Life after Distal Radius Fractures. Rehabil. Med..

[9] Golec P., Depukat P., Rutowicz B., Walocha E., Mizia E., Pełka P., Kopacz P., Tomaszewski K.A., Walocha J. (2015). Main Health-Related Quality-of-Life Issues in Patients after a Distal Radius Fracture. Folia Med. Cracov..

[10] Morasiewicz P., Zaborska M., Sobczak M., Tomczyk Ł., Leyko P., Bobiński A., Kochańska-Bieri J., Pili D., Kazubski K. (2024). The Use of Osteogenon as an Adjunctive Treatment in Lower Leg Fractures. Pharmaceuticals.

[11] Varga O.I., Vezikova N.N., Marusenko I.M., Kheĭfets L.M. (2007). Osteogenon in Therapy of Distal Radial Bone Fractures in Patients with Secondary Osteoporosis. Ter. Arkhiv.

[12] Morasiewicz P., Zaborska M., Sobczak M., Tomczyk Ł., Pili D., Kazubski K., Leyko P. (2025). The Use of Ossein–Hydroxyapatite Complex in Conjunction with the Ilizarov Method in the Treatment of Tibial Nonunion. J. Clin. Med..

[13] Klum M., Wolf M.B., Hahn P., Leclère F.M., Bruckner T., Unglaub F. (2012). Normative Data on Wrist Function. J. Hand Surg. Am..

[14] Schmidt V., Gordon M., Petterson A., Buttazzoni C., Seimersson A., Sayed-Noor A., Mukka S., Wadsten M. (2024). Functional Outcomes Are Restored a Decade after a Distal Radius Fracture: A Prospective Long-Term Follow-up Study. J. Hand Surg..

[15] Olech J., Konieczny G., Tomczyk Ł., Morasiewicz P. (2021). A Randomized Trial Assessing the Muscle Strength and Range of Motion in Elderly Patients Following Distal Radius Fractures Treated with 4- and 6-Week Cast Immobilization. J. Clin. Med..

[16] Bobos P., Lalone E.A., Grewal R., MacDermid J.C. (2018). Do Impairments Predict Hand Dexterity After Distal Radius Fractures? A 6-Month Prospective Cohort Study. HAND.

[17] Venkatesh R.B. (2016). A Comparative Study between Closed Reduction and Cast Application Versus Percutaneous K- Wire Fixation for Extra- Articular Fracture Distal End of Radius. J. Clin. Diagn. Res..

[18] Testa G., Vescio A., Di Masi P., Bruno G., Sessa G., Pavone V. (2019). Comparison between Surgical and Conservative Treatment for Distal Radius Fractures in Patients over 65 Years. J. Funct. Morphol. Kinesiol..

[19] Kim T.S., Park D.D.H., Lee Y.B., Han D.G., Shim J.s., Lee Y.J., Kim P.C.W. (2014). A Study on the Measurement of Wrist Motion Range Using the IPhone 4 Gyroscope Application. Ann. Plast. Surg..

[20] Morley J.E. (2014). Is It Possible to Prevent Injurious Falls?. Eur. Geriatr. Med..

[21] Melzer I., Freiberger E., Britting S., Lattanzio F., Melzer Y., Ben-Romano R., Roller-Wirnsberger R., Wirnsberger G., Mattace-Raso F., Tap L. (2025). Characteristics of Falls among Community-Dwelling Older Adults: The SCOPE Study. Gerontology.

[22] Coviello M., Abate A., Ippolito F., Nappi V., Maddalena R., Maccagnano G., Noia G., Caiaffa V. (2022). Continuous Cold Flow Device Following Total Knee Arthroplasty: Myths and Reality. Medicina.

[23] Heyer F.L., de Jong J.J.A., Willems P.C., Arts J.J., Bours S.G.P., van Kuijk S.M.J., Bons J.A.P., Poeze M., Geusens P.P., van Rietbergen B. (2021). The Effect of Bolus Vitamin D3 Supplementation on Distal Radius Fracture Healing: A Randomized Controlled Trial Using HR-pQCT. J. Bone Miner. Res..

[24] Aspenberg P., Genant H.K., Johansson T., Nino A.J., See K., Krohn K., García-Hernández P.A., Recknor C.P., Einhorn T.A., Dalsky G.P. (2010). Teriparatide for Acceleration of Fracture Repair in Humans: A Prospective, Randomized, Double-Blind Study of 102 Postmenopausal Women with Distal Radial Fractures. J. Bone Miner. Res..

[25] Crandall C.J., Larson J., Shadyab A.H., LeBoff M.S., Wactawski-Wende J., Weitlauf J.C., Saquib N., Cauley J.A., Saquib J., Ensrud K.E. (2024). Physical Function Trajectory after Wrist or Lower Arm Fracture in Postmenopausal Women: Results from the Women’s Health Initiative Study. Osteoporos. Int..

[26] Veronese N., Ragusa F.S., Sabico S., Dominguez L.J., Barbagallo M., Duque G., Al-Daghri N. (2024). Osteosarcopenia Increases the Risk of Mortality: A Systematic Review and Meta-Analysis of Prospective Observational Studies. Aging Clin. Exp. Res..

[27] Parimon T., Cusack B., Rea I.M., Carvalho A. (2014). Physical Activity and Cognitive Function in Individuals over 60 Years of Age: A Systematic Review. Clin. Interv. Aging.

[28] Nguyen A., Vather M., Bal G., Meaney D., White M., Kwa M., Sungaran J. (2020). Does a Hand Strength–Focused Exercise Program Improve Grip Strength in Older Patients With Wrist Fractures Managed Nonoperatively?. Am. J. Phys. Med. Rehabil..

[29] Hopewell S., Chan A.W., Collins G.S., Hróbjartsson A., Moher D., Schulz K.F., Tunn R., Aggarwal R., Berkwits M., Berlin J.A. (2025). CONSORT 2025 Statement: Updated guideline for reporting randomised trials. BMJ.

